# Supplementary material for: Ultrasensitive Stress Biomarker Detection Using Polypyrrole Nanotube Coupled to a Field-Effect Transistor
Source: Micromachines (Basel). 2020 Apr 22;11(4):439. doi: 10.3390/mi11040439 (PMC7231345; doi:10.3390/mi11040439)
Supplement: Supplementary file 1 [file micromachines-11-00439-s001.pdf]

micromachines

Supplementary Materials:

Ultrasensitive Stress Biomarker Detection Using Polypyrrole Nanotube Coupled to a Field-effect Transistor

Kyung Ho Kim [1,2,†], Sang Hun Lee [3,†], Sung Eun Seo [1], Joonwon Bae [4], Seon Joo Park [1,*] and Oh Seok Kwon [1,*]

[1] Infectious disease Research Center, Korea Research Institute of Bioscience and Biotechnology (KRIBB), Daejeon 34141, Korea; doublekh0119@gmail.com (K.H.K.); eun93618@kribb.re.kr (S.E.S.)
[2] Department of Chemical Engineering and Applied Chemistry, Chungnam National University, Daejeon 305-764, Korea
[3] Department of Bioengineering, University of California Berkeley, Berkeley, CA 94720, USA; shlee.ucb@gmail.com
[4] Department of Applied Chemistry, Dongduk Women's University, Seoul 02748, Korea; redsox7@dongduk.ac.kr
* Correspondence: seonjoopark86@kribb.re.kr (S.J.P.); oskwon79@kribb.re.kr (O.S.K.); Tel.: +82-42-860-8284 (O.S.K.)
† These authors contributed equally to this work.

Received: 21 March 2020; Accepted: 21 April 2020; Published: 22 April 2020 date

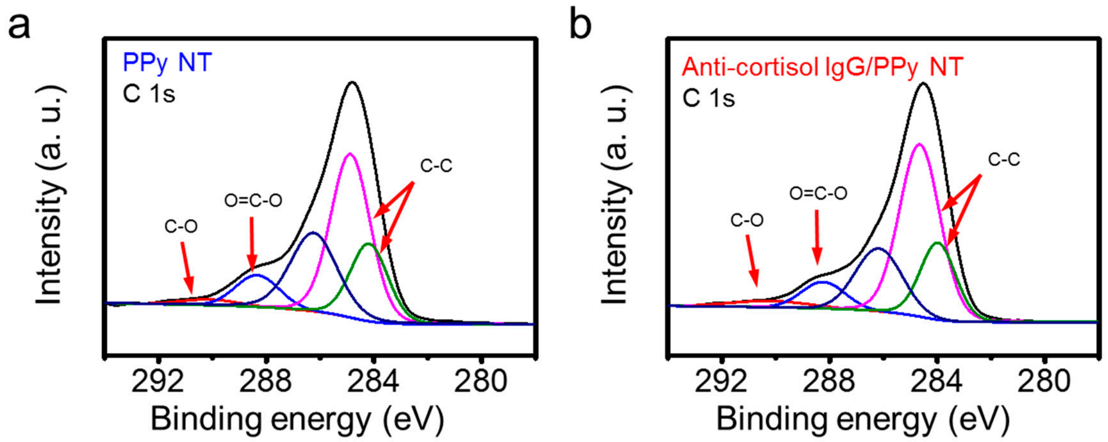

Figure S1. Measured and fitted C *1s* XPS narrow spectrum of (**a**) PPy NT and (**b**) Anti-cortisol IgG/PPy NT, respectively.

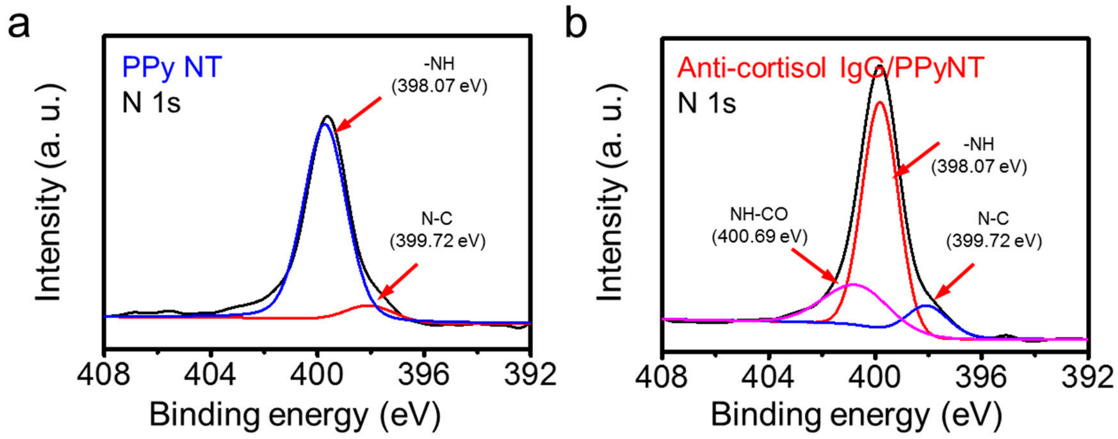

Figure S2. Measured and fitted XPS spectra. The N *1s* narrow spectrum of (**a**) PPy Ny and (**b**) Anti-cortisol IgG/PPy NT.

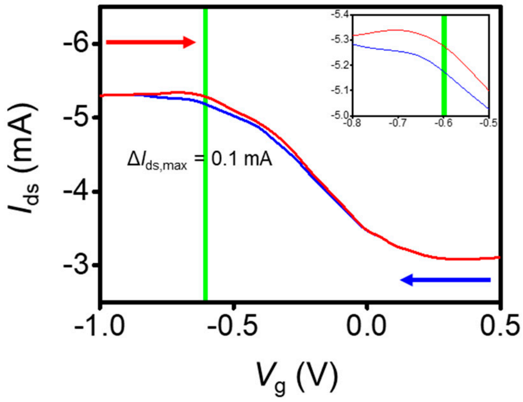

Figure S3. Typical transfer curve for hysteresis confirmation measured at V_{ds} = −1 mV and between the cyclic sweeps.

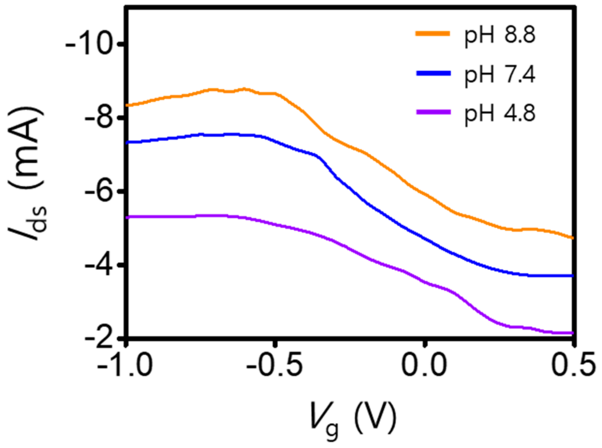

Figure S4. The transfer curve of anti-cortisol IgG/PPyNT FET depending on pH effect (pH 4.8, pH 7.4 and pH 8.8).

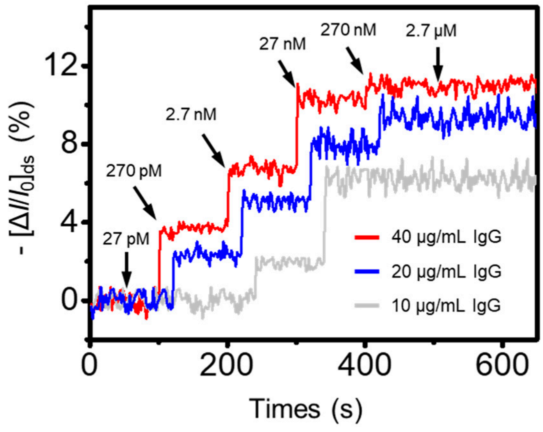

Figure S5. The real-time response of depending on the anti-cortisol concentrations (10, 20 and 40 μg/mL).

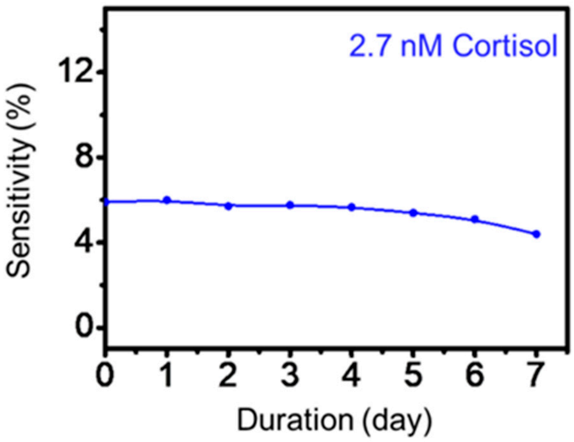

Figure S6. Life span test of our sensor platform. Cortisol with 2.7 nM was stimulated for 7 days to evaluate their storage stability.

© 2020 by the authors. Licensee MDPI, Basel, Switzerland. This article is an open access article distributed under the terms and conditions of the Creative Commons Attribution (CC BY) license (http://creativecommons.org/licenses/by/4.0/).
